# Congenital Aortico-Left Ventricular Tunnel: A Case Report of a Rare Cause of Aortic Regurgitation in Adults

**Published:** 2017-10

**Authors:** Zahra Khajali, Sedigheh Saedi, Alireza Alizadeh Ghavidel, Hamid Reza Pouraliakbar

**Affiliations:** Department of Cardiology, Rajaie Cardiovascular, Medical, and Research Center, Iran University of Medical Sciences, Tehran, Iran

**Keywords:** *Aortic valve insufficiency*, *Heart defects, congenital*, *Congenital abnormalities*

## Abstract

The aortico-left ventricular tunnel is a rare congenital abnormality resulting in a pathologic connection between the aorta and the left ventricle. It often presents during infancy or early childhood as a cardiac failure symptom or an incidental finding of a cardiac murmur due to severe aortic regurgitation. It is, however, also occasionally found in asymptomatic adults. We describe a 20-year-old female presenting with palpitations in whom clinical evaluations with echocardiography and computed tomography angiography led to the diagnosis of severe aortic regurgitation caused by a tunnel connecting the right sinus of the aorta to the left ventricle. The patient underwent successful obstruction of the tunnel with an autologous pericardial patch and the repair of the dilated aortic root via the reduction aortoplasty technique. She was discharged on the 5th postoperative day with no complications. At 1 month’s follow-up, she remained asymptomatic and echocardiography showed aortic valve competence with no residual regurgitation.

## Introduction

Aortic regurgitation or an abnormal backflow of blood into the left ventricle (LV) during diastole may result from such diverse pathologies as diseases of the aortic valve leaflets and the aortic root. Isolated or primary congenital aortic valve regurgitation is rather uncommon and is generally due to anatomically anomalous valves including bicuspid, quadricuspid, fenestrated, dysplastic, and absent aortic valves as well as the aortico-LV tunnel.^1^^-^^3^ Here we report a rare presentation of congenital, isolated, and severe aortic regurgitation in an adult patient resulting from an abnormal communication between the aorta and the LV.

## Case Report

A 20-year-old female presented to our clinic complaining of frequent palpitations. Her past medical history was unremarkable, and she mentioned occasional consumption of propranolol for her palpitations. 

**Figure 1:**
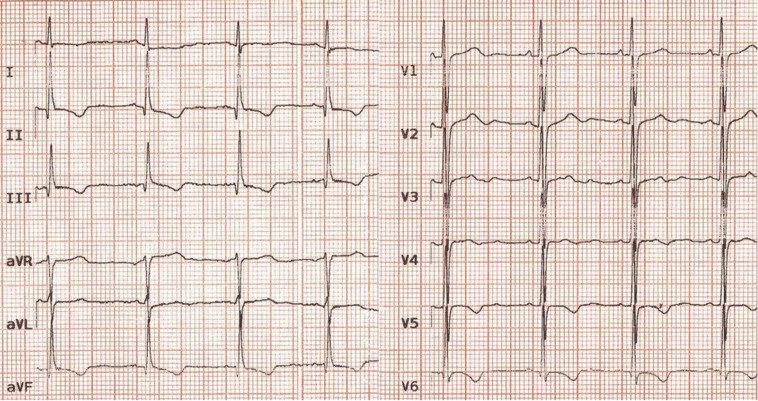
Twelve-lead electrocardiography of the patient, showing a strain pattern.

**Figure 2:**
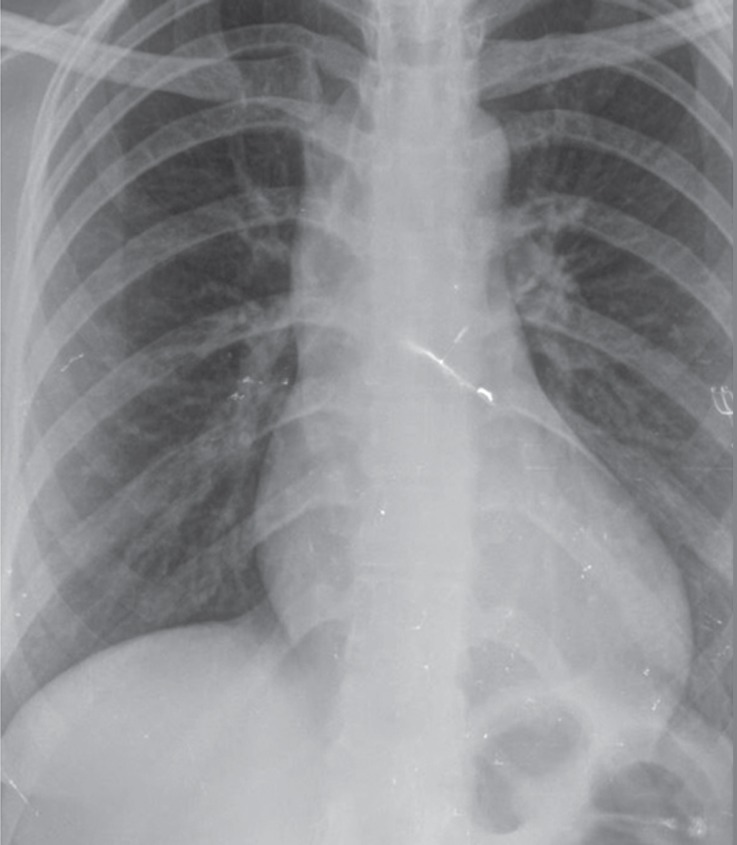
Chest X-ray (posteroanterior view) of the patient, depicting cardiomegaly.

The physical examination revealed a diastolic murmur (grade III/VI), best heard at the left sternal border. The electrocardiography (ECG) showed normal sinus rhythm, mild LV hypertrophy, and strain pattern in the inferior and precordial leads (Figure 1). The chest X-ray depicted a mildly increased cardiothoracic ratio, in favor of LV enlargement (Figure 2).

**Figure 3:**
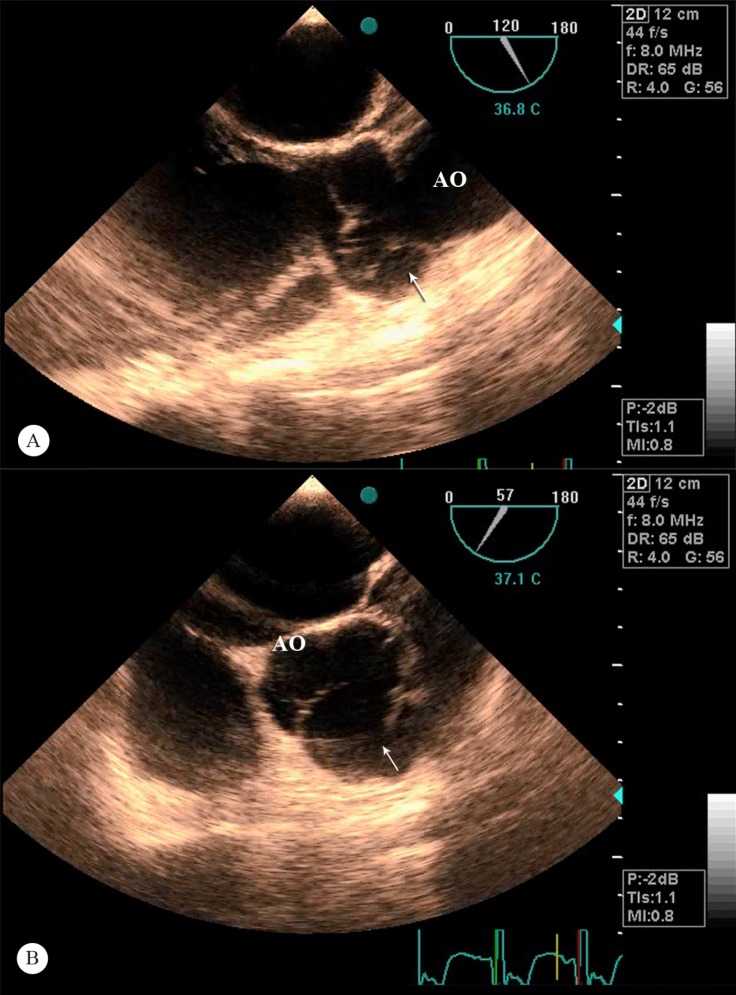
Transesophageal echocardiographic long axis (A) and short-axis (B) views of the aortico-left ventricular tunnel (Arrow) and the relation to the aorta (AO).

The patient underwent transthoracic and transesophageal echocardiography, which revealed a dilated right sinus of Valsalva (4.2 cm) and severe aortic regurgitation through a defect originating above the aortic root and the right coronary cusp with termination into the LV outflow tract (Figure 3). There was also moderate central aortic regurgitation and no sign of a dissection flap. The LV size was severely enlarged, but the LV systolic function was preserved (ejection fraction = 50%) 

**Figure 4:**
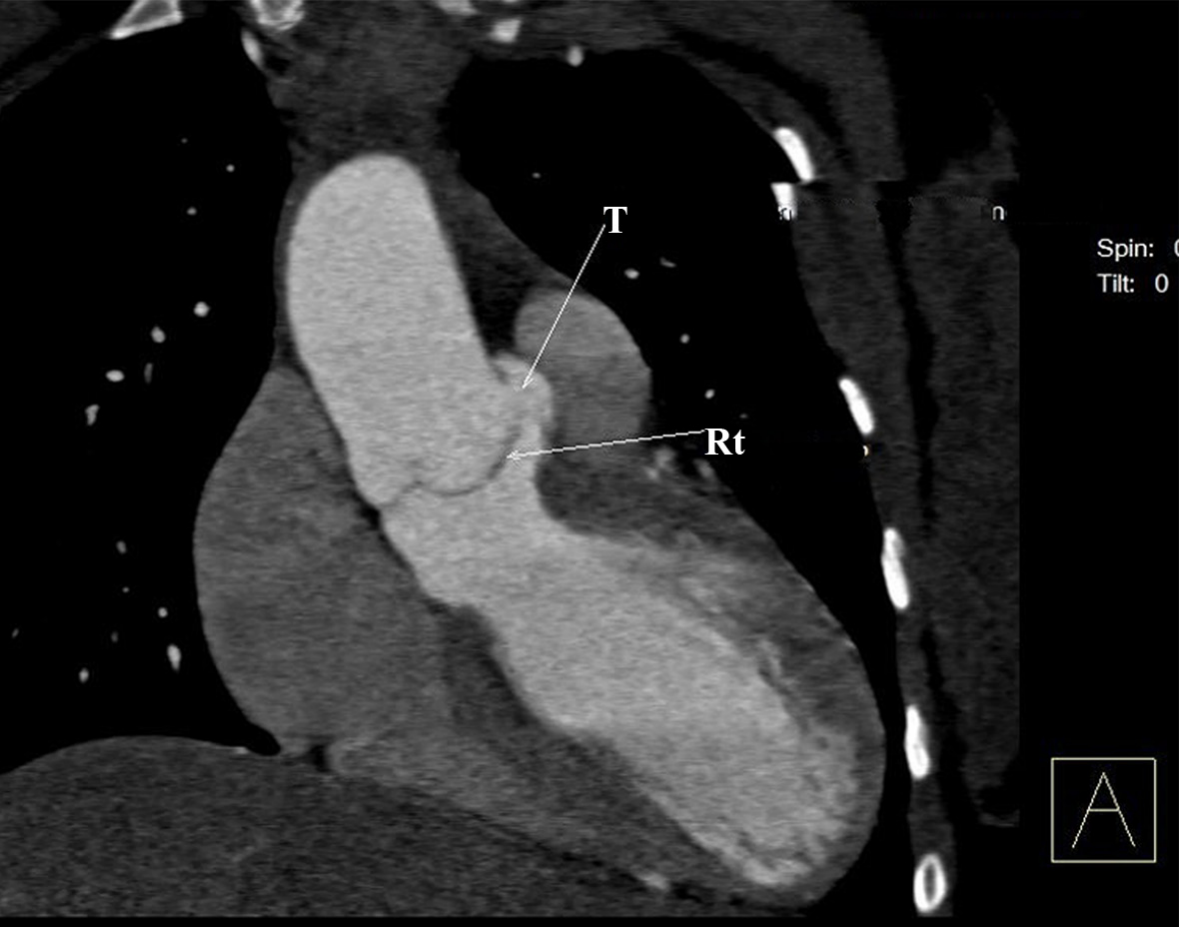
Computed tomography angiography, showing the aortico-left ventricular tunnel (T).

Cardiac computed tomography angiography confirmed the diagnosis of an aortico-LV tunnel by depicting the entry of the contrast material from an opening above the right sinus of Valsalva into the LV, with no evidence of a ruptured sinus of Valsalva aneurysm or aortic dissection (Figure 4). The course of the coronary arteries was also normal.

Rt, Right coronary cusp of aorta

**Figure 5:**
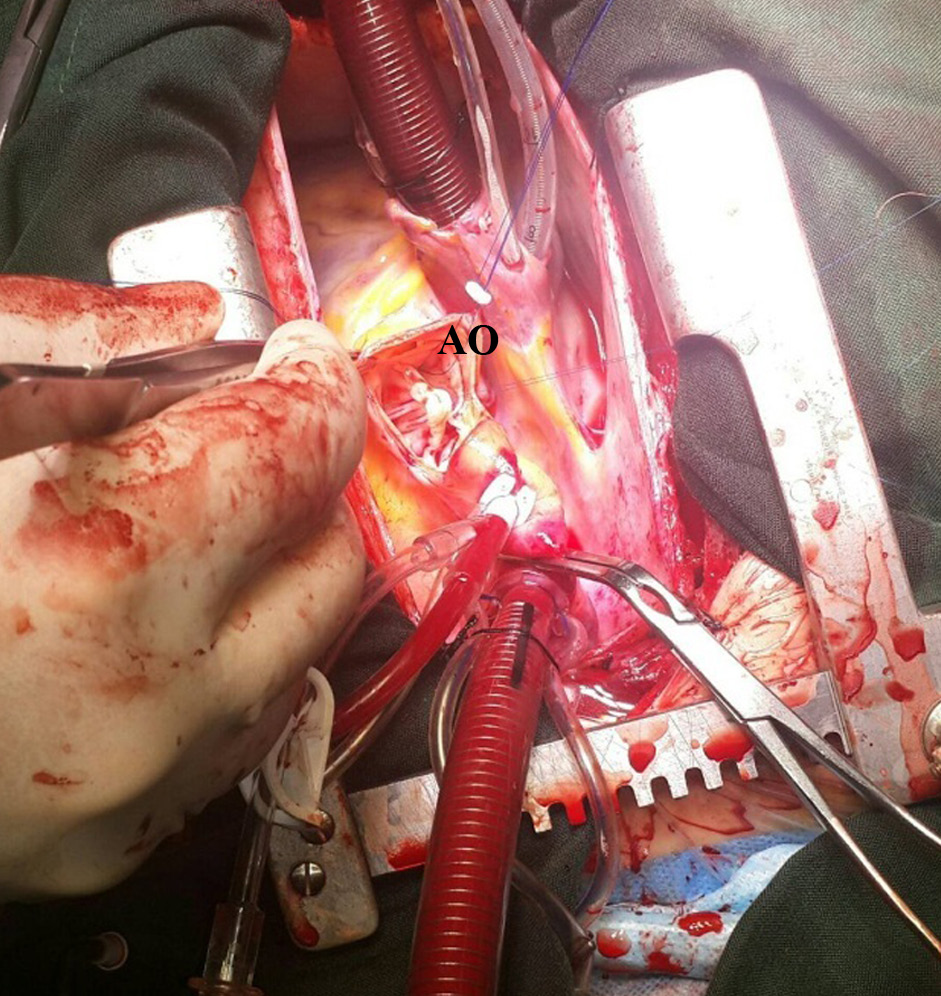
Operative repair of the aortico-left ventricular tunnel.

The patient was scheduled for cardiac surgery. Under general anesthesia, median sternotomy was performed. Cardiopulmonary bypass with mild hypothermia was established after heparin administration (3 mg/kg) and standard ascending aorta and right atrium cannulation. Cardiac arrest was induced using a cold blood cardioplegic solution via antegrade and retrograde routes. After classic aortotomy, the aortic valve was evaluated and the aortic site of the tunnel was identified. This anomaly had created a 12 mm slit-like defect along two-thirds of the right coronary leaflet hinge from the commissure of the right and left coronary leaflets to the nadir of the right cusp. The margin of the defect was sharp without any gross evidence of endocarditis or degenerative changes. In addition, there was a small fenestration on the free margin of the right coronary leaflet. The defect was repaired with a non-treated autologous pericardial patch using a double-ended 6-0 Prolene suture, and the free margin fenestration was repaired with a figure-of-eight 6-0 Prolene suture. Then the reductive annuloplasty was done with 3 separated 4-0 horizontal mattress pledgeted Prolene sutures on each commissure. The patient was weaned form cardiopulmonary bypass without inotropic support, and the post-pump intraoperative transesophageal echocardiography showed trivial aortic regurgitation and a preserved LV function (Figure 5). The postoperative course was uneventful except for episodes of supraventricular tachycardia, which were managed medically. At 1-month’s follow-up, the patient was well and asymptomatic. 

AO, Aorta

## Discussion

The embryologic etiology of the aortico-LV tunnel remains controversial but the abnormal development of a tissue plane separating the aortic and pulmonary valves has been indicated.^4^^, ^^5^ This abnormality is very rare with an estimated incidence of 0.001% of congenital heart diseases and about 130 cases have been reported in the literature so far.^5^ In this anomaly, an abnormal paravalvular tunnel-like route originating from the ascending aorta bypasses the aortic valve and opens into the LV cavity just below the coronary cusps. Most commonly the right coronary sinus is involved; however, the origin of the tunnel above the left and noncoronary sinuses has also been reported.^6^^, ^^7^ Associated abnormalities include coronary artery anomalies and aortic or pulmonary valvular disease.^8^ Patients usually present at infancy or early childhood with congestive heart failure symptoms or dyspnea on exertion. Very uncommonly this condition is encountered in adults who are mostly asymptomatic or have minimal clinical symptoms. Echocardiography is a valuable tool for initial diagnosis in that it shows the origin of the aortic regurgitation and the orifice of the tunnel above the aortic sinus. An important differential diagnosis is the sinus of Valsalva aneurysm and rupture, in which the opening is within the aortic sinus.^5^ Cardiac computed tomography angiography or magnetic resonance imaging provides a higher spatial resolution and may be useful in differentiating these anomalies with greater anatomical details.^9^ Although there are case reports of percutaneous device closure of the defect, surgery is the ideal and standard treatment strategy and should be performed as soon as the diagnosis is made even in asymptomatic patients to prevent further myocardial damage and LV dilation.^4^^, ^^5^^, ^^10^ The surgical technique should include the closure of both ends of the tunnel with a pericardial patch and primary sutures based on the patient’s condition and the surgeon’s discretion. Direct suturing of the orifices might increase the aortic cusp deformity and lead to valvular regurgitation. The possibility of coronary artery damage due to the abnormal coronary course through the tunnel should also be considered before corrective surgery.

After the surgery, close clinical follow-up is needed as the recurrence of aortic regurgitation remains a postsurgical concern with a reported prevalence of 16% to 60% and the need for aortic valve replacement of up to 50%.^7^

## Conclusion

Our patient was one of rarely reported adults diagnosed with an aortico-LV tunnel as the cause of her severe aortic regurgitation, resulting in LV enlargement and dysfunction necessitating surgical correction. This report also emphasizes the need for a thorough and detailed search for the mechanism of valvular abnormalities in young patient populations with the advent of recent technological advances in the field of cardiac imaging.

## Notes:

***This paper should be cited as: ***Khajali Z, Saedi S, Alizadeh Ghavidel A, Pouraliakbar HR. Congenital Aortico-Left Ventricular Tunnel: A Case Report of a Rare Cause of Aortic Regurgitation in Adults. J Teh Univ Heart Ctr 2017;12(4):167-170.
